# Bilateral angle closure glaucoma with retinitis pigmentosa in young patients: case series

**DOI:** 10.1186/s12886-023-03190-y

**Published:** 2023-11-15

**Authors:** Ziyang Lu, Lu Wang, Xi Ying, Lian Tan

**Affiliations:** 1 Southwest Hospital / Southwest Eye Hospital, Third Military Medical University (Army Medical University), Chongqing, 400038 China; 2 Key Lab of Visual Damage and Regeneration & Restoration of Chongqing, Chongqing, 400038 China; 3 Department of Ophthalmology, the 958Th Hospital, Southwest Hospital, Third Military Medical University (Army Medical University), Chongqing, 400038 China

**Keywords:** Retinitis pigmentosa, Primary angle closure glaucoma, *ZNF408*

## Abstract

**Background:**

To report the ocular characteristics and management of three cases of retinitis pigmentosa (RP) concurrent primary angle closure glaucoma (PACG).

**Case presentation:**

Three middle-aged patients presenting with diminished vision, high intraocular pressure (IOP), and typical fundus manifestations of RP were clinically evaluated. The individualized treatment was based on the ocular conditions of each case. A novel genetic alteration in *ZNF408* was identified in one patient. Two patients with short-axial eyes received unilateral combined trabeculectomy, cataract surgery, and Irido-zonulo-hyaloid-vitrectomy. One of them had a subluxated lens, managed with a capsular tension ring implantation. Their contralateral eyes, respectively, underwent laser peripheral iridotomy (LPI) and transscleral cyclophotocoagulation. The third patient underwent bilaterally combined laser peripheral iridoplasty, LPI, and medication. Ultimately, all patients achieved the target IOP during a two-year follow-up.

**Conclusion:**

Young patients with RP may have a risk of developing angle closure glaucoma, and conversely, patients with angle closure glaucoma at younger age should be aware of the presence of RP. Therefore, routine gonioscopy and IOP monitoring are required for RP patients, and detailed fundus examinations are warranted for young PACG patients.

**Supplementary Information:**

The online version contains supplementary material available at 10.1186/s12886-023-03190-y.

## Background

Retinitis pigmentosa (RP), a leading cause of human blindness worldwide, is an inherited retinal degenerated disease [1]. It features progressive loss of photoreceptors and retinal pigment epithelium (RPE). The fundus appearances manifest as waxy optic discs, attenuated retinal vessels, and peripheral pigment migration resembling bony spicules [2]. The full-field electroretinogram (ff-ERG), an essential diagnostic tool for RP, indicates a reduction in a- and b-wave amplitudes. The disease is typically characterized by nyctalopia, decreased visual acuity, and gradual visual field defect, with or without abnormal color vision [1]. The possible explanation of reduced vision in RP can be progressive photoreceptor cell death and treatable complications, including cataract, macular abnormalities, glaucoma, and so on. The worldwide prevalence of RP is about 1 in 4000 for a total of more than 1 million patients affected. In previous clinical practices, the co-occurrence of RP and glaucoma has been reported since 1862 [3]. A population-based case–control study in Taiwan showed that RP patients had a 3.64-fold greater odds of developing acute angle closure (ACG) than in the general population [4], supporting a strong association between RP and angle closure glaucoma.

Here, we report three cases of primary angle closure glaucoma (PACG) in RP along with their personalized treatments. Informed consent was obtained from the patients, and all the work conducted was in accordance with the Declaration of Helsinki.

## Case presentations

### Case 1

**Fig. 1:**
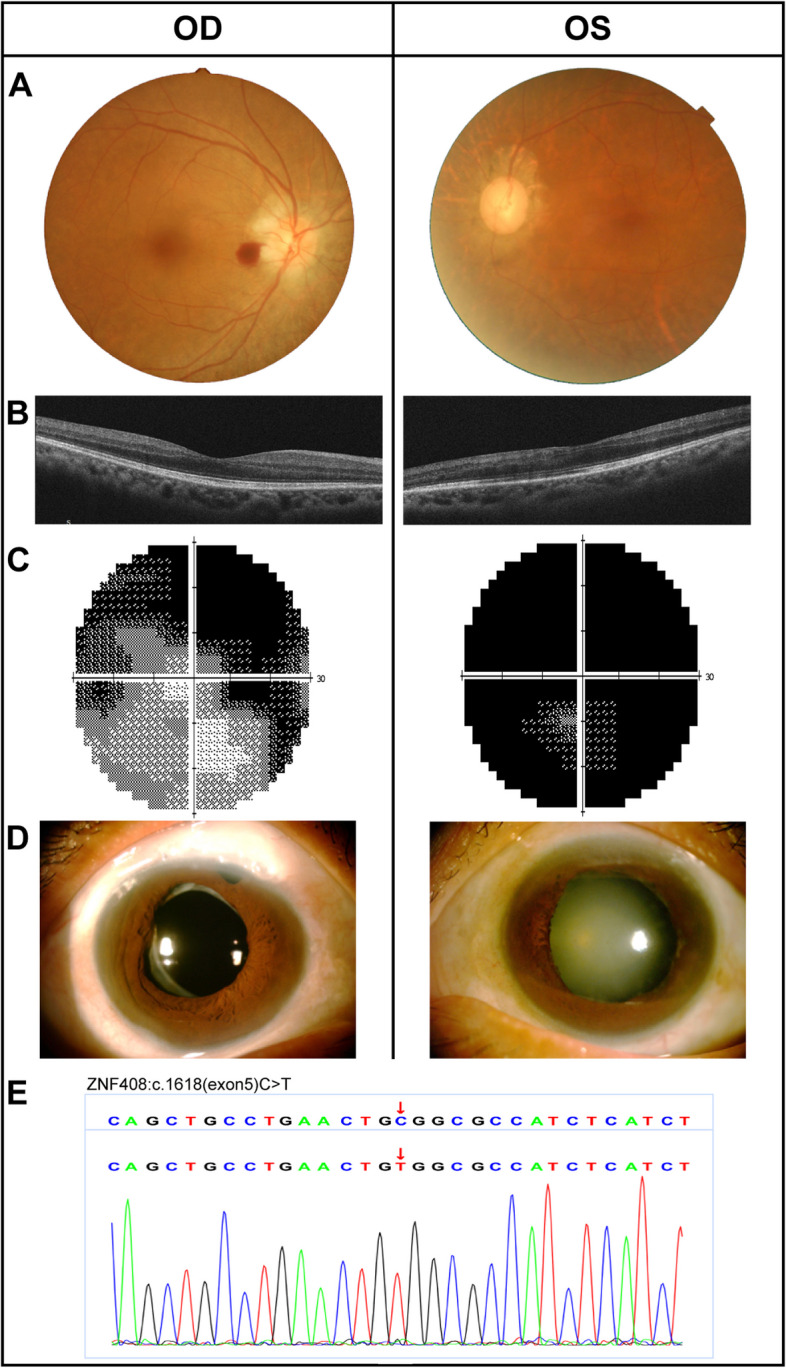
Clinical examinations of Case 1. **A** The fundus photography showing a disc hemorrhage OD and a cup to disc ratio of 0.5 OD and 0.6 OS. **B** Macular OCT suggesting no significant abnormality OU. **C** A visual field with retention of a small central and inferotemporal island OD and no residual visual field OS were observed. **D** The anterior segment photograph at 1-year follow-up revealing a functional filtering bleb OD, IOL in place OD, and normal anterior chamber OU. **E** Partial sequence diagram of *ZNF408* exon 5. A homozygous mutation (c.1618 C > T transition)

A 53-year-old female presented with 10 days history of blurry vision in the left eye. She had a family history of RP. On examination, the best-corrected visual acuity (BCVA) was 20/63 OD and only light perception OS. Intraocular pressure (IOP) was 19 mmHg OD and 33 mmHg OS for the first visit, and rising to 27 mmHg OD and 37 mmHg OS one week later, respectively. Pigmentary keratic precipitates (KP), Tyndall phenomenon, dilated pupils with no light response, narrow peripheral anterior chamber (PAC) with Van Herrick (VH) grade 1, and slight lens opacification were observed in bilateral eyes. The presence of osteoblast-like pigmentation in the inferior peripheral retina OU was observed (Fig. S1). The cup to disc ratio (CDR) was nearly 0.5 OD and 0.6 OS (Fig. 1A). No significant abnormalities were evident on the macular optical coherence tomography (OCT) of both eyes (Fig. 1B). The right eye had a visual field defect with retention of a small central and inferotemporal island, while no residual visual field existed in the left eye (Fig. 1C). Gonioscopy of both eyes revealed iridotrabecular synechia in full-circumferential range. A remarkable reduction in a- and b-wave amplitudes was seen in ff-ERG (Fig. S2). We applied genetic testing to the patient and identified a novel single nucleotide homozygous, transversion mutation (c.1618 C > T) of exon 5 in the *ZNF408* gene (Fig. 1E). Both eye received two times daily topical beta-blocker (0.5% timolol), two times daily carbonic anhydrase inhibitors (0.15% brinzolamide), three times daily topical alpha-2 agonists (0.2% brimonidine tartrate) and one times daily travoprost (0.004%). But the left eye still had uncontrolled IOP and consequently undergoing transscleral cyclophotocoagulation (TCP). IOP in the right eye remained well-controlled with topical anti-glaucoma medications for ten months, and became elevated and progressively worsened to a peak of 43 mmHg. Hence, combined cataract surgery, trabeculectomy and the Irido-zonulo-hyaloid-vitrectomy (IZHV) were performed in the right eye. The postoperative anterior segment was kept in a stable condition (Fig. 1D), with BCVA improving to 20/28 OD, and IOP was maintained normal during a 2-year follow-up.

### Case 2

**Fig. 2:**
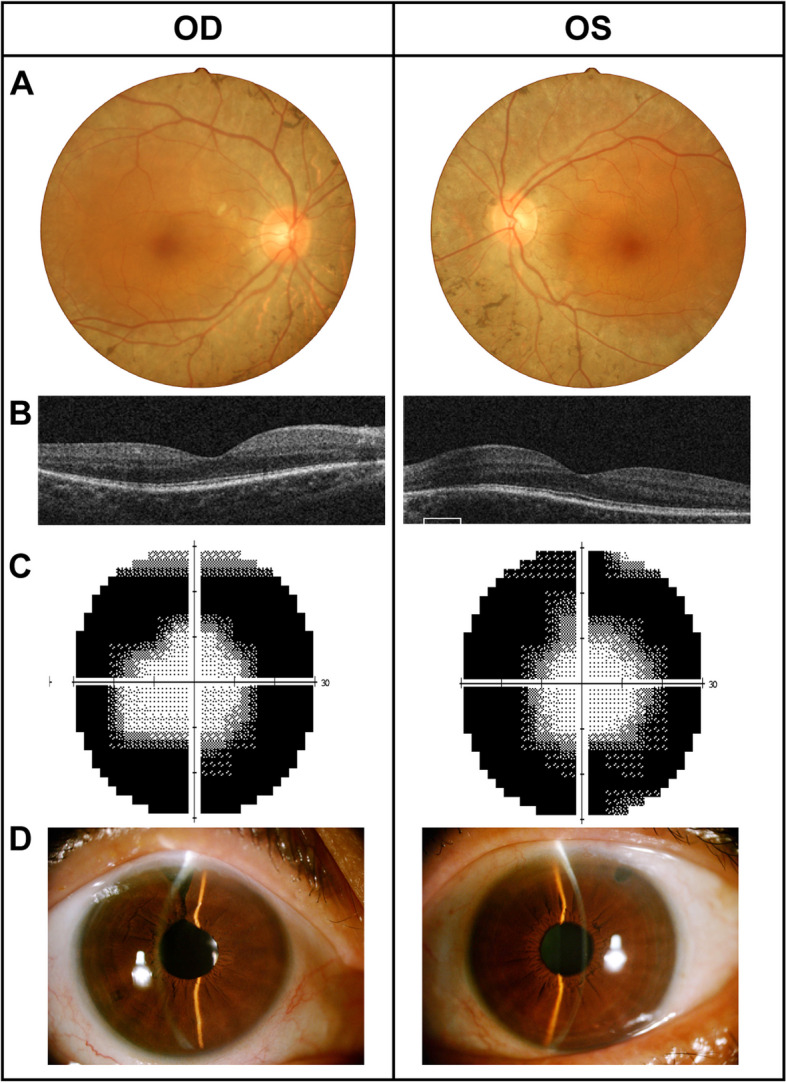
Clinical examinations of Case 2. **A** Fundus photography of both eye showing waxy pale optic discs and retinal hyperpigmentation in bone spicules at the mid-periphery and periphery retina. **B** Macular OCT showing no significant abnormality OU. **C** Visual field testing demonstrating residual tubular visual field in both eyes. **D** Anterior segment photography at postoperative year 1 revealing a deep anterior chamber of both eyes

A 54-year-old female presented with a 10 days history of painless visual loss in the right eye. She was otherwise systemically well and had no significant family history. The BCVA was 20/80 OD and 20/40 OS. Goldmann applanation tonometry (GAT) revealed an IOP of 38 mmHg OD and 14 mmHg OS. The depth of PAC was VH grade 1 OU. The pupil was mid-dilated and minimally reacting to light OD. The lens was slightly opacity OU. A fundus examination revealed that discs were waxy pale with a CDR of 0.4 OD and 0.3 OS, respectively. Bony spicule pigmentation diffused in the mid-peripheral and peripheral retina (Fig. 2A). Macular OCT suggested no significant abnormality OU (Fig. 2B). However, a tubular visual field was observed in bilateral eyes (Fig. 2C). Decreased a- and b-wave amplitudes were observed in both eyes with ff-ERG (Fig. S2). Gonioscopy revealed closed angle in all quadrant OD, whereas narrow ACA in three quadrants OS. The ocular biometric parameters of both eyes were shown in Table 1. The IOP was out of control with four kinds of anti-glaucoma medications, thus combined cataract surgery and trabeculectomy were performed on the right eye. Whole-circumference unstable zonular fibers were discovered during phacoemulsification, treated by the implantation of a capsular tension ring. Simultaneously, the right eye underwent the IZHV to prevent malignant glaucoma. Laser peripheral iridotomy (LPI) was performed in the left eye (Fig. 2D). The postoperative BCVA was 20/32 OD, and IOP was maintained within normal at postoperative 2 years.

**Table 1:** Clinical characteristics and management of the three cases in the study

	**Age (yrs)**	**Gender**		**AL (mm)**	**LT (mm)**	**ACD (mm)**	**Presenting BCVA**	**Presenting IOP (mmHg)**	**Management**	**2-year postoperative BCVA**	**2-year postoperative IOP (mmHg)**
**Case 1**	53	Female	OD	21.78	5.27	1.73	20/63	19	Trab + Phaco + IOL + AV	20/28	8.6
			OS	21.80	5.25	1.64	LP	33	TCP	LP	12.4
**Case 2**	54	Female	OD	21.75	4.85	1.61	20/80	38	Trab + Phaco + IOL + CTR + AV	20/32	13
			OS	21.62	4.64	2.50	20/40	14	LPI	20/40	17
**Case 3**	47	Male	OD	26.63	5.21	2.09	20/125	27	LPIp + LPI	20/200	9.4
			OS	26.34	5.06	2.23	20/32	27	LPIp + LPI	20/40	9.4

*AL* Axial length, *LT* Lens thickness, *ACD* Anterior chamber depth, *BCVA* Best Corrected Visual Acuity, *LP* light perception, *Trab* trabeculectomy, *Phaco* phacoemulsification, *IOL* intraocular lens, *TCP* transscleral cyclophotocoagulation, *CTR* capsular tension ring, *AV* anterior vitrectomy, *LPI* laser peripheral iridotomy, *LPIp* laser peripheral iridoplasty

### Case 3

**Fig. 3:**
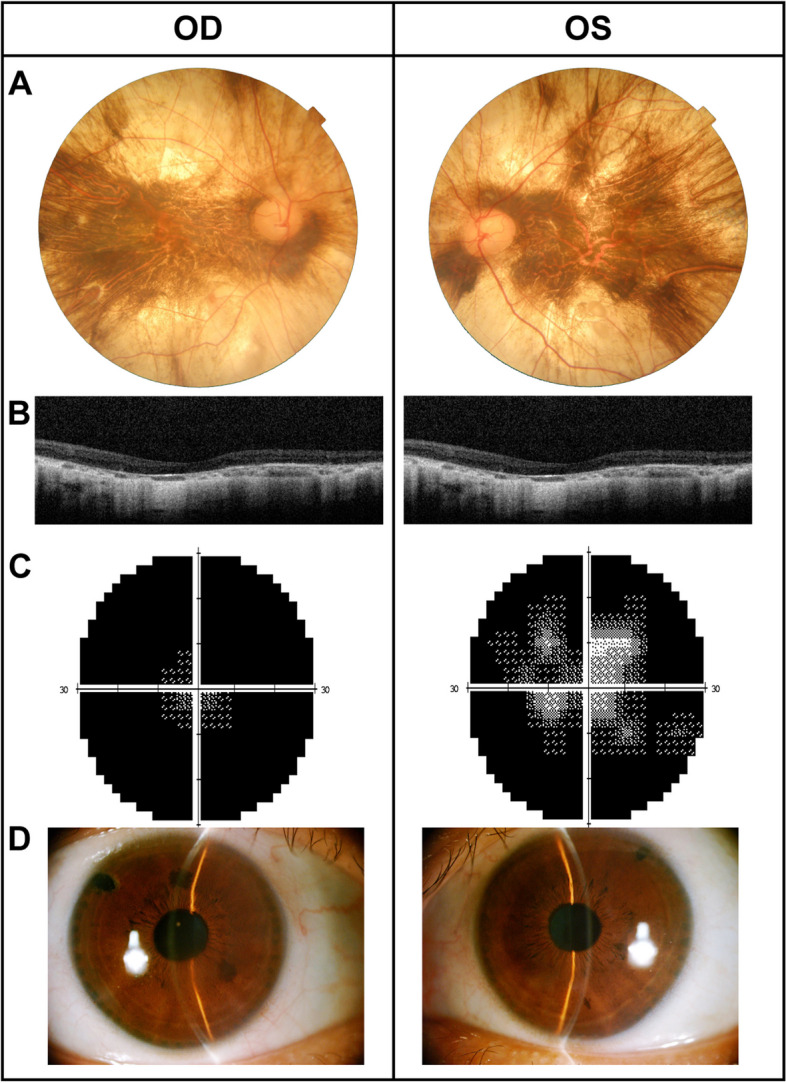
Clinical examinations of Case 3. **A** The fundus photography of both eyes with advanced retinitis pigmentosa showing narrowed retinal vessels, extensive chorioretinal atrophy, and waxy pallor of optic discs. **B** Macular OCT of both eyes identifying severe outer retinal layer thinning. **C** Visual field with retention of a small central island was detected in bilateral eyes. **D** Anterior segment photography at postoperative year 1 revealing a wide peripheral anterior chamber bilaterally

A 47-year-old male with established RP for 13 years was found high IOP bilaterally on the regular reviews. He had a history of high myopia. The BCVA was 20/125 OD and 20/32 OS. IOP with applanation was 27 mmHg OU. A slit-lamp examination showed the peripheral anterior chamber of both eyes was shallow with VH grade 2. The fundus examination revealed the retinal vessels were strongly attenuated, diffused chorioretinal atrophy OU and waxy pale discs with a CDR of 0.9 OD and 0.8 OS. We scarcely found bony spicules existed in the retina (Fig. 3A) and no detectable waves in ff-ERG (Fig. S2). Macular OCT revealed an attenuated outer nuclear layer, RPE, and loss of outer/inner segments (Fig. 3B). Significantly great impairment was observed with perimetry (Fig. 3C). Gonioscopy bilaterally indicated the four-quadrant angle was iridotrabecular appositonal and could be re-opend in dynamic gonioscope, so combined laser peripheral iridoplasty and LPI were performed in both eyes. The postoperative PAC widened (Fig. 3D) and IOP consistently was kept under control throughout a 2-year follow-up with the application of topical IOP-lowering eye drops.

## Discussion and conclusions

In this series, we described three uncommon cases of RP concomitant PACG attending our hospital in 2020 and performed a regular follow-up. In our previous work, RP patients from January 2014 to January 2019 were studied retrospectively, which found concurrent glaucoma accounting for 1.6% [5]. Moreover, we reviewed the prevalence of PACG in RP patients, and figured out the Asians appear to rank the highest, especially the Chinese (Table 2). This might be explained by a larger sample size of RP and a higher prevalence in China, approximately 1.6–4.0 times than worldwide [1, 6, 7]. Furthermore, nanophthalmos, cataract, lens subluxation, increased lens thickness (LT) and anterior segment dysgenesis in RP were predisposing factors for ACG anatomically [8, 9]. As shown in Table 1, two patients had short axial eyes (Axial length < 22 mm). Zonular instability and thicker lens were also observed among them.

**Table 2:** Comparison of the prevalence of PACG in RP patients in previous studies

**First author (Year of publication)**	**District**	**Subjects (No.)**	**PACG (Subjects /%)**	**Age**^a^
**D W Peng (1991) **[10]	China	1400	30 (2.14%)	44.0 (range 13–63)
**O Badeeb (1993) **[11]	Canada	538	5 (0.93%)	57.2 (range 32–74)
**Ko YC (2014) **[4]	Taiwan, China	382	5 (1.3%)	53.3 ± 8.0
**Pradhan C (2020) **[12]	Nepal	234	5 (2.13%)	56.6 ± 8.3
**Wang DD (2022) **[13]	China	1356	39 (2.88%)	46.04 ± 14.50
**Hung MC (2022) **[14]	Taiwan, China	6223	100 (1.61%)	49.0 ± 18.1
**Pradhan ZS (2022) **[15]	India	618	14 (2.27%)	52.7 ± 2.4

^a^The average age of the confirmed diagnosis of PACG in RP patients

Prevalence of PACG increased with aging steadily. People older than 80 years old have highest prevalence of 2.8%, while people aged 40–49 years have the lowest prevalence of 0.1% [16]. RP patients had an earlier onset of PACG (Table 2) [4, 10–15]. The average age at which PACG is diagnosed in the general population is (34.4 ± 5.1) years old, while that in RP patients is (29.7 ± 7.0) years old [9]. This was contrary to the fact that PACG was more prevalent in the elderly population [4, 8, 16]. Meanwhile, the incidence of PACG in Chinese RP patients (Table 2) was higher than that in the general middle-aged and elderly (40 years old and above) population [16]. Those findings may suggest that a higher risk of developing angle closure glaucoma exist in younger patients with retinitis pigmentosa. However, more than half of the RP patients co-occurred with PACG had a normal optic disc [10]. A tubular visual field might be observed in the patients of RP or PACG at the late stage. It is worth noting that atypical glaucomatous optic discs, similar visual defects, and the incidence of rarity may lead to a higher misdiagnosis rate. Hence, comprehensive ophthalmic examinations, including gonioscopy, ff-ERG, macular OCT and genetic testing, could be warranted in younger RP patients in clinical diagnosis.

Zhong et al.’s exploration using whole exome sequencing discovered that five genes, *CRB1、COL2A1、RHO、RP1L1* and *PAX6*, were responsible for the genetic association between early-onset PACG and RP [17, 18]. Therein, *CRB1* and *RHO* had been implicated in familial forms of nanophthalmos [19–22]. *COL2A1* and *PAX6* were pivotal regulators of anterior segment dysgenesis [23–25]. To our knowledge, here we first report *ZNF*408 as a new gene associated with RP concurrent PACG, which was only previously identified alone in familial exudative vitreoretinopathy or RP [26, 27]. *ZNF408* encodes a zinc finger protein that harbors 10 C2H2-type fingers thought to be implicated in DNA binding. ZNF408 is mostly expressed in human retinas, including photoreceptors, amacrine, ganglion cells and retinal blood vessels [26]. Its mutation could lead to impairment of visual function and typical changes of RP [26]. Remarkably, this is also the first report that the *ZNF408* mutation was identified domestically in RP patients.

The current therapeutic strategies for PACG comprised laser, medication, or surgery (trabeculectomy, lens extraction, combined lens extraction with trabeculectomy, goniosynechialysis, etc.). The management based on cases were listed in Table 1. Notably, two patients underwent anterior vitrectomy. Prior clinical studies demonstrated that the anterior chamber tended to become refractory shallow in PACG patients with retinal disorders after trabeculectomy [28, 29]. Moreover, choroidal expansion secondary to fundus vascular abnormalities, shorter AL and zonular slackness had been proven to increase the incidence of postoperative malignant glaucoma [30, 31]. IZHV has been applied by many ophthalmologists as a golden standard to manage malignant glaucoma [32]. It was also noteworthy that two middle-aged patients without significant lens opacities underwent cataract surgeries. Our previous studies showed that cataract was the second complication in RP patients in Western China, representing approximately 43.1% [5]. Researches concerning RP unambiguously pointed out that inflammation occupied a crucial position in disease progression [33–35]. Persistent ocular inflammation supposedly damaged lens zonules and induced lens opacity, leading to severe vision impairment and anterior chamber angle occlusion. Therefore, the timing for cataract surgery was recommended to be appropriate earlier for RP patients to alleviate vision impairment and monitor the fundus changes [36–38]. All three patients achieved satisfactory outcomes in a two-year follow-up.

In conclusion, through describing three cases with PR concomitant PACG and their corresponding clinic treatments, we supposed that young patients with RP might have a risk of developing angle closure glaucoma, and conversely, patients with angle closure glaucoma at younger age should be aware of the presence of RP.

### Supplementary Information

**Additional file 1: Supplementary Figure 1.** The fundus photography of Case 1 showed bony spicule pigmentary changes at the inferior peripheral retina (red rectangle). **Supplementary Figure 2.** The full-field electroretinogram(ff-ERG) of three cases. Scotopic and photopic responses with a slight reduction in the amplitude of a and b waves were observed in Case 1, while nondetectable waves were in Case 2.  A remarkable reduction in a- and b-wave amplitudes were seen in Case 3.

## Data Availability

Data could be available from the corresponding author by reasonable inquire.

## Abbreviations

RP: Retinitis pigmentosa
PACG: Primary angle-closure glaucoma
IOP: Intraocular pressure
LPI: Laser peripheral iridotomy
RPE: Retinal pigment epithelium
ff-ERG: Full-field electroretinogram
PAC: Peripheral anterior chamber
OCT: Optical coherence tomography
ACA: Anterior chamber angle
TCP: Transscleral cyclophotocoagulation
IZHV: Irido-zonulo-hyaloid-vitrectomy
GAT: Goldmann applanation tonometry
AL: Axial length
LT: Lens thickness
ACD: Anterior chamber depth
BCVA: Best Corrected Visual Acuity
LP: Light perception
Trab: Trabeculectomy
Phaco: Phacoemulsification
IOL: Intraocular lens
CTR: Capsular tension ring
AV: Anterior vitrectomy
LPIp: Laser peripheral iridoplasty
