# Supplementary material for: Bilateral angle closure glaucoma with retinitis pigmentosa in young patients: case series
Source: BMC Ophthalmol. 2023 Nov 15;23:458. doi: 10.1186/s12886-023-03190-y (PMC10648655; doi:10.1186/s12886-023-03190-y)
Supplement: Supplementary file 1 — Additional file 1: Supplementary Figure 1. The fundus photography of Case 1 showed bony spicule pigmentary changes at the inferior peripheral retina (red rectangle). Supplementary Figure 2. The full-field electroretinogram(ff-ERG) of three cases. Scotopic and photopic responses with a slight reduction in the amplitude of a and b waves were observed in Case 1, while nondetectable waves were in Case 2. A remarkable reduction in a- and b-wave amplitudes were seen in Case 3. [file 12886_2023_3190_MOESM1_ESM.docx]

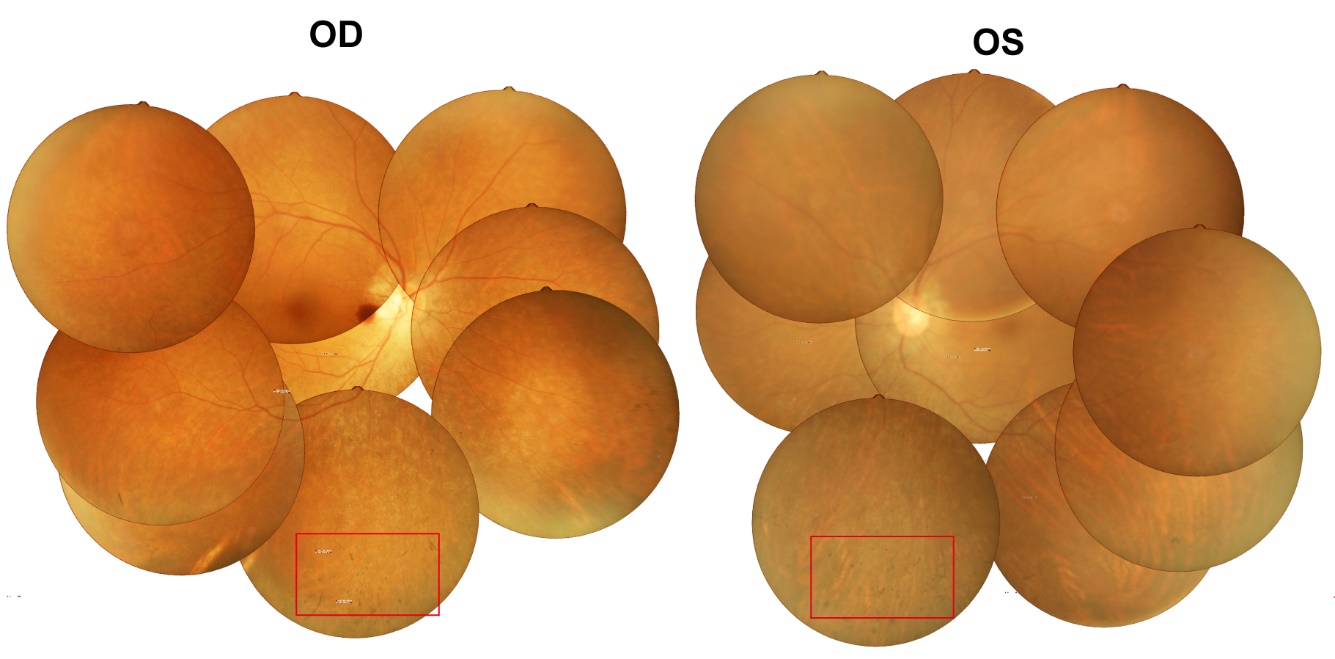


**Supplementary Figure 1. The fundus photography of Case 1 showed bony spicule pigmentary changes at the inferior peripheral retina (red rectangle).**


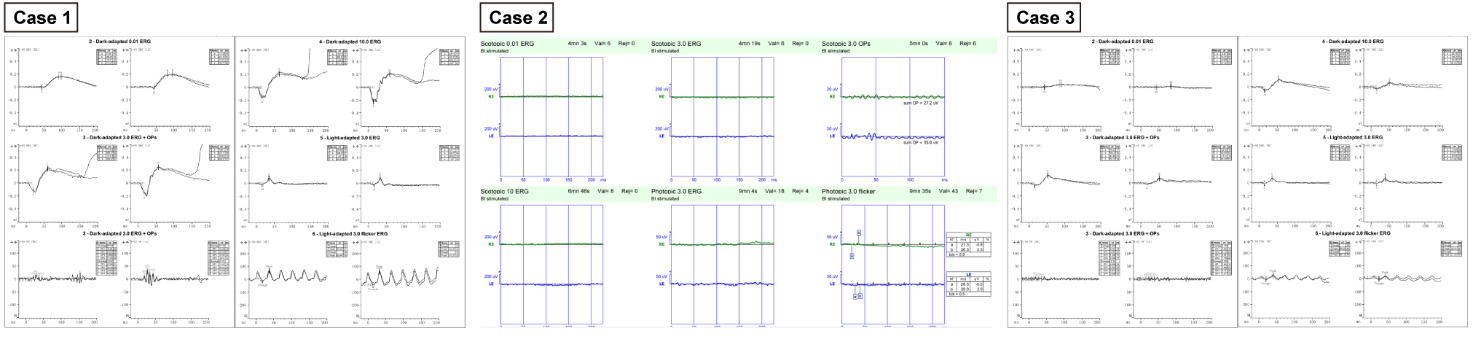


**Supplementary Figure 2. The full-field electroretinogram(ff-ERG) of three cases.** Scotopic and photopic responses with a slight reduction in the amplitude of a and b waves were observed in Case 1, while nondetectable waves were in Case 2.  A remarkable reduction in a- and b-wave amplitudes were seen in Case 3.
